# Induced Cell Cycle Arrest in Triple-Negative Breast Cancer by Combined Treatment of Itraconazole and Rapamycin

**DOI:** 10.3389/fphar.2022.873131

**Published:** 2022-04-19

**Authors:** Hua-Tao Wu, Chun-Lan Li, Ze-Xuan Fang, Wen-Jia Chen, Wen-Ting Lin, Jing Liu

**Affiliations:** ^1^ Department of General Surgery, The First Affiliated Hospital of Shantou University Medical College, Shantou, China; ^2^ Guangdong Provincial Key Laboratory for Diagnosis and Treatment of Breast Cancer, Cancer Hospital of Shantou University Medical College, Shantou, China; ^3^ Department of Physiology/Changjiang Scholar’s Laboratory, Shantou University Medical College, Shantou, China; ^4^ Department of Pathology, Shantou University Medical College, Shantou, China

**Keywords:** itraconazole, rapamycin, triple-negative breast cancer, cell cycle, apoptosis

## Abstract

Triple-negative breast cancer (TNBC) is the aggressive molecular type of breast carcinoma, with a high metastasis/relapse incidence and cancer-related death rate, due to lack of specific therapeutic targets in the clinic. Exploring potential therapeutic targets or developing novel therapeutic strategies are the focus of intense research to improve the survival and life quality of patients with TNBC. The current study focused on drugs targeting the mTOR signaling pathway by investigating the potential utilization of itraconazole (ITZ) combined with rapamycin in the treatment of TNBC. CCK-8, colony formation and transwell assays were conducted to evaluate the effect of ITZ with rapamycin in combination on MDA-MB-231 and BT-549 TNBC cells. Synergistic inhibition was found in terms of proliferation and motility of TNBC cells. However, apoptosis was not enhanced by the combined treatment of ITZ and rapamycin. Flow cytometry analysis showed that ITZ and/or rapamycin arrested cells in G0/G1 phase and prevented G1/S phase transition. Reduced cyclin D1 protein levels were consistent with G0/G1 phase arrest, especially when resulting from the combination of ITZ with rapamycin. In conclusion, the combination of ITZ with rapamycin is a promising therapeutic strategy for patients with TNBC through synergistically arresting cells in the G0/G1 phase of the cell cycle, rather than inducing apoptosis.

## Introduction

Itraconazole (ITZ), a broad-spectrum antifungal agent, has recently been verified as an anti-cancer drug in preclinical and clinical research (Pantziarka et al., 2015). ITZ exerts its antifungal activity through decreasing ergosterol synthesis, which is required for the membrane integrity of fungal cells (Poulsen et al., 2021). Based on its antifungal effects, ITZ is used as a safe and effective long-tern prophylaxis for fungal infections in immunocompromised cancer patients with neutropenia (Muhldorfer and Konig, 1990).

The effects and mechanisms of action for ITZ’s anti-cancer activities have been reported in different types of cancer *in vitro* and *in vivo*. Its anti-cancer potential to reverse multi-drug resistance (MDR) has been shown in daunorubicin-resistant P388 leukemia cells (Gupta et al., 1991), adriamycin-resistant K562 leukemia cells (Kurosawa et al., 1996), human breast cancer resistance protein-expressing HEK cells resistant to topotecan (Gupta et al., 2007), P-glycoprotein (MDR1)-overexpressing, multidrug-resistant HeLa cells (Iida et al., 2001), bevacizumab-resistant gastrointestinal cancer (Hara et al., 2016) and metastatic castration-resistant prostate cancer (Antonarakis et al., 2013). It has also been reported that ITZ inhibits tumor growth and angiogenesis or induces apoptosis and autophagy in non-small cell lung cancer (NSCLC) (Aftab et al., 2011; Gerber et al., 2020), gastric cancer (Hu et al., 2017; Lan et al., 2018), cervical cancer (Rojo-Leon et al., 2019), and skin cancer (Liang et al., 2017; Carbone et al., 2018). Importantly, the underlying molecular mechanism of ITZ’s anti-cancer effect has been reported *via* inhibiting hedgehog signaling, Wnt pathway and/or reducing mTOR expression, recognized as an mTOR inhibitor (Head et al., 2015; Wang et al., 2020).

Growing evidence demonstrates that combined therapeutic strategies with ITZ are promising for cancer patients. Chemotherapy with ITZ has been reported as a promising treatment for patients with unresectable gastric cancer (Sawasaki et al., 2020). A prospective randomized controlled study found that the utilization of ITZ in patients with NSCLC significantly benefits patients in terms of 1-year progression-free survival and overall response rate, although no improvement was found in terms of 1-year overall survival (Mohamed et al., 2021). As arsenic trioxide and ITZ antagonize the hedgehog pathway, Ally *et al.* proposed a sequential arsenic trioxide and ITZ treatment for metastatic basal cell carcinoma and found that the combined treatment is a feasible strategy for metastatic basal cell carcinoma patients (Ally et al., 2016). For colorectal cancer, ITZ synergistically increases the therapeutic effect of paclitaxel and ^99m^Tc-methoxyisobutylisonitrile accumulation in an HT-29 human colorectal tumor-bearing animal model (Ghadi et al., 2021).

Breast cancer, the most common female malignant tumor, threatens the patients’ health worldwide (Siegel et al., 2021). Among the different molecular types of breast cancer, triple-negative breast cancer (TNBC) is highly aggressive and has a high incidence of metastasis/relapse and increased mortality due to the absence of the estrogen receptor (ER), progesterone receptor (PR) and human epidermal growth factor receptor 2 (HER2) expression, hindering the ability to employ targeted therapy (Cao et al., 2021). Interestingly, for heavily treated patients with TNBC, especially patients with recurring tumors, chemotherapy with ITZ has shown a promising effect in the clinic (Tsubamoto et al., 2014). To evoke drug repurposing and drug combinations, a screening of the most promising drugs with verapamil and ITZ was conducted and identified the combination of ITZ and 5-fluorouracil as the most effective by decreasing cell viability and proliferation (Correia et al., 2018). Recently, EI-Sheridy *et al.* used miltefosine-modified lipid nanocapsules to develop an ITZ nanoformulation, with a relatively small size and high entrapment efficiency, to enhance the chemotherapeutic efficacy of ITZ, regarding inhibition of tumor growth and cellular proliferation (El-Sheridy et al., 2021). Based on the above evidence, ITZ shows promise as an anti-cancer drug in the treatment of patients with TNBC.

Head *et al.* focused on the molecular mechanism of ITZ and found the suppressive function of ITZ on the AMPK/Mtor signaling axis (Head et al., 2015). The mTOR signaling pathway is essential for cell proliferation and survival, and in many types of cancers, mTOR is typically abnormally activated due to metabolic changes or mutations in upstream regulatory factors. Not surprisingly, inhibition of the mTOR pathway is a promising strategy for the development of anticancer drugs and has been demonstrated in clinical studies (Ferrari et al., 2022). Rapamycin, another antifungal drug extracted from Streptomyces hygroscopicus, is currently considered as a potent immunosuppressant in clinical and a target for mTOR, a serine/threonine protein kinase (Sabatini, 2017), exerting cytotoxicity against various kinds of cancers (Jelonek et al., 2021). However, Lee *et al.* demonstrated that rapamycin did not inhibit the activation of downstream effectors of mTOR completely (Lee et al., 2021), leading to the compensatory up-regulation of AKT activity and chemotherapy, limiting the utilization of rapamycin as an independent therapy (Mossmann et al., 2018).

So, the current study targets the AKT/mTOR signaling pathway, downstream of ITZ (Head et al., 2015), investigates the anti-cancer effect of monotherapy using ITZ and another drug each, as well as the two in combination, in TNBC cells. Also, the potential molecular mechanism of the therapeutic effect was also evaluated to provide potential drug combinations for patients with TNBC.

## Materials and Methods

### Chemical Reagents

ITZ (HY-17514) and rapamycin (HY-10219) were purchased from MedChemExpress (United States).

### Cell Culture

The human triple-negative breast cancer cell lines MDA-MB-231 and BT-549 were from the cell bank of the Chinese Academy of Sciences (Shanghai). MDA-MB-231 cells were cultured in Dulbecco’s modified Eagle’s medium plus 10% fetal bovine serum (FBS, GIBCO, Brazil) and 1% penicillin-streptomycin antibiotics (Beyotime, China). BT-549 cells were cultured in RPMI 1640 medium (GIBCO, Brazil) plus 10% FBS and 1% penicillin-streptomycin. All cells were cultured in a 37°C incubator containing 5% carbon dioxide.

### CCK-8 Assay

Determination of IC50 concentration: 5 × 10^3^ cells were inoculated into each well of a 96-well plate. After 24 h, culture medium containing a series of drug dilutions was changed and cells were further cultured for 48 h. Then, 10 µL CCK-8 reagent (Invigentech, United States) and 100 µL culture medium were added to each well and cells were cultured an additional 2 h before absorbance was measured at 450 nm. Growth inhibition was calculated as Inhibition rate% = (OD value of control group-OD value of experimental group)/(OD value of control group-OD value of the blank) × 100%. The SPSS 19.0 software was used for statistical analysis of data to obtain half maximal growth inhibitory concentration (IC50) value of drugs, each experiment was repeated for three times.

Quantitation of cell proliferation: cells were inoculated at 1 × 10^3^ cells per well into 96-well plates and cultured at 37C, 5% CO_2_ and allowed to attach for 24 h. Drugs at their half maximal growth inhibitory concentration (IC50) concentrations were added. After 24, 48, 72, 96, and 120 h, 10 μL CCK-8 reagent and 100 μL medium were added for 2 h at 37°C. Then absorbance of each well was measured at 450 nm using a spectrophotometer.

### Colony Formation

Cells were inoculated into a 60 mm dish with drug at the IC50 concentration for 48 h. Then, cells were resuspended and seeded at 1 × 10^3^ cells per well in 6-well plates and cultured for 14 days. Cells were fixed with methanol and stained with 0.1% crystal violet. Count the size and number of cell colonies in each group.

### Transwell Assay

Chambers with 8-µM pore membranes were used for migration assays. Cells were initially cultured for 48 h in media without and with different concentrations of drugs. Then, cells were digested with pancreatin and diluted in serum-free medium and inoculate into the upper transwell chambers at a density of 2 × 10^4^ cells per chamber. Complete medium was added to the bottom chamber. After 24 or 48 h, migrated cells were stained with 0.1% crystal violet.

### Flow Cytometry

For quantitation of apoptosis, Annexin V-FITC Detection Kit (C1063, Beyotime, China) was used and cells were inoculated into a 60 mm dish with drugs at IC50 concentrations for 48 h. After washing the cells twice with PBS, the cells were digested and resuspended in binding buffer containing 5 µL of an Annexin V-FITCAfter incubation at room temperature for 20 min, 10 µL PI was added to the suspension and incubated for another 10 min. Then the degree of apoptosis was detected by flow cytometry (Accuri C6, Becton-Dickinson, United States).

For quantitation of cell cycle phases, Cell cycle Detection Kit (BestBio, China) was applied and cells were incubated with drugs for 24 h, then digested and collected into 70% ethanol at -20°C and allowed to fix for 2 h. The fixed cells were centrifuged at 1000 rpm and then washed with PBS, resuspended in staining buffer (0.5 ml) containing PI (25 µL) and RNase A (10 µL), and incubated at 37°C for 30 min in the dark. Finally, the DNA level was measured by flow cytometry.

### Western Blotting

Cell cultures were incubated in 60 mm dishes with different concentrations of drug for 48 h and then washed twice with PBS for digestion and cell collection. The cells were lysed for 30 min in ice-cold RIPA buffer with phenylmethylsulfonyl fluoride and Protein phosphatase inhibitor (Solarbio, China). Then, lysates were ultrasonicated three times (4 s each) and centrifuged at 12,000 rpm for 15 min to remove the precipitate, and the protein concentration was determined using a bicinchoninic acid assay (BCA) kit (GenStar, China). After denaturation, 30 µg of protein was subjected to SDS-polyacrylamide gel electrophoresis, transferred to a polyvinylidene fluoride membrane, and blocked with 5% milk. The PVDF membrane was incubated with primary antibodies overnight to detect the expression of mTOR pathway-related proteins and cycle-related proteins. Primary antibodies and diluted concentration were followed: Akt (1:1000, #4691, CST, United States), p-Akt (1:1000, #4060, CST, United States), mTOR (1:1000, #2983, CST, United States), p-mTOR (1:1000, #5536, CST, United States), p70S6K (1:1000, #9202, CST, United States), p-p70S6K (1:1000, #9205, CST, United States), cyclin D1 (1:1000, #2978, CST, United States), GAPDH (1:1000, #TA309157, ZSGB-BIO, China), p21 (1:1000, #2947, CST, United States), β-Actin (1:1000, #TA-09, ZSGB-BIO, China). After incubation with primary antibody, membranes were washed, then incubated with HRP-conjugated secondary antibody (1:2000) at room temperature for 120 min. Enhanced chemiluminescence chemicals were used to visualize target proteins and chemiluminescence western blotting detection system (Bio-Rad ChemiDoc XRS+, United States) was used for protein detection.

## Results

### Itraconazole Inhibits the Proliferation and Motility of Triple-Negative Breast Cancer Cells Through Suppressing the AKT/mTOR Pathway

**FIGURE 1:**
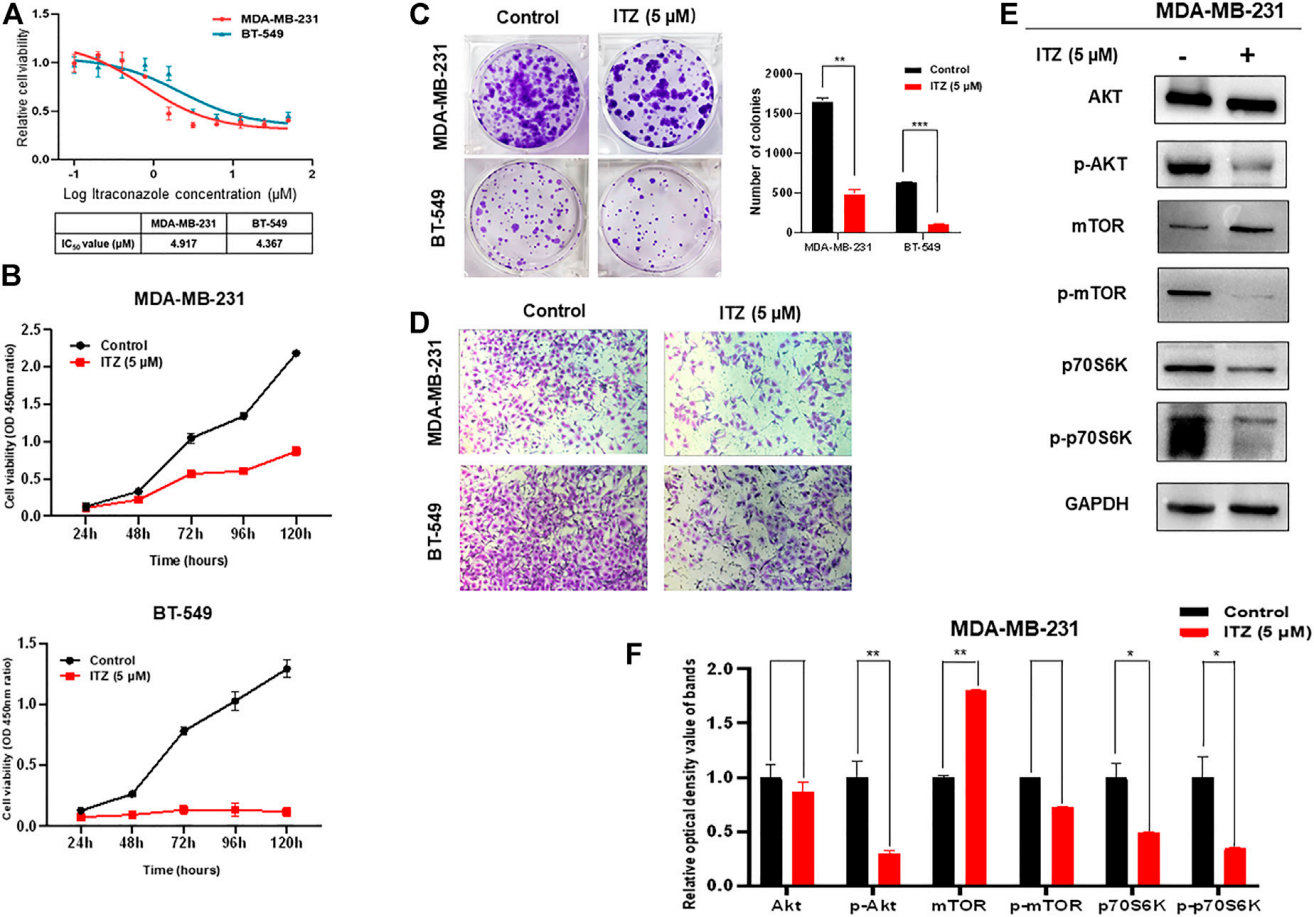
Effect of ITZ on TNBC cells. **(A)** Proliferation of TNBC cells was dose-dependently inhibited by varying concentrations of ITZ **(B)** The proliferation rate of TNBC cells was suppressed by ITZ in time-dependent manner **(C)** Colony formation assay showing ITZ treatment reduces both the number and size of colonies formed by both MDA-MB-231 and BT-549 cells, quantified and analyzed in histogram **(D)** Transwell assay showing the motility of MDA-MB-231 and BT-549 cells was dramatically inhibited by ITZ treatment **(E,F)** Activity of the AKT/mTOR signaling pathway was decreased by ITZ treatment. The quantified results from Western blot **(E)** were analyzed with statistical significance in histogram **(F)**. *p < 0.05, **p < 0.01, ***p < 0.001.

To investigate the potential function and mechanism of ITZ in TNBC, the concentration of ITZ required for treating TNBC cells was determined by CCK-8 assay to identify IC50 for MDA-MB-231 and BT-549 cells. Results showed that with increasing concentration of ITZ, the survival rate of TNBC cells decreased in a dose-dependent manner (Figure 1A), with IC50s of 4.917 μM for MDA-MB-231 and 4.367 μM for BT-549 obtained.

The anti-cancer activities of ITZ were examined by measuring the effects of ITZ on the proliferation and motility of TNBC cells. In the CCK-8 assay, TNBC cells were exposed with or without ITZ at the IC50 concentration for 24, 48, 72, 96, and 120 h. ITZ treatment inhibited the proliferation of both MDA-MB-231 and BT-549 cells (Figure 1B). Colony formation was also decreased by ITZ, compared with the control group, in both MDA-MB-231 and BT-549 cells (Figure 1C). Furthermore, transwell assays suggested that treatment with ITZ suppressed the migratory ability of MDA-MB-231 and BT-549 cells (Figure 1D).

To explore the underlying mechanism of ITZ in TNBC cells, protein levels of AKT/mTOR signaling pathway components were examined by western blotting. Interestingly, the activated forms of AKT and mTOR were decreased with ITZ treatment, while the expression levels of AKT and mTOR were not suppressed in the ITZ-treated groups. To confirm the involvement of the AKT/mTOR signaling pathway, phosphorylation of the mTOR downstream target p70S6K was also examined. Phosphorylation of p70S6K was inhibited in the ITZ-treated groups, consistent with reduced p-AKT and p-mTOR levels (Figures 1E,F).

### Inhibition of the AKT/mTOR Pathway by Rapamycin Inhibits the Proliferation and Motility of Triple-Negative Breast Cancer Cells

**FIGURE 2:**
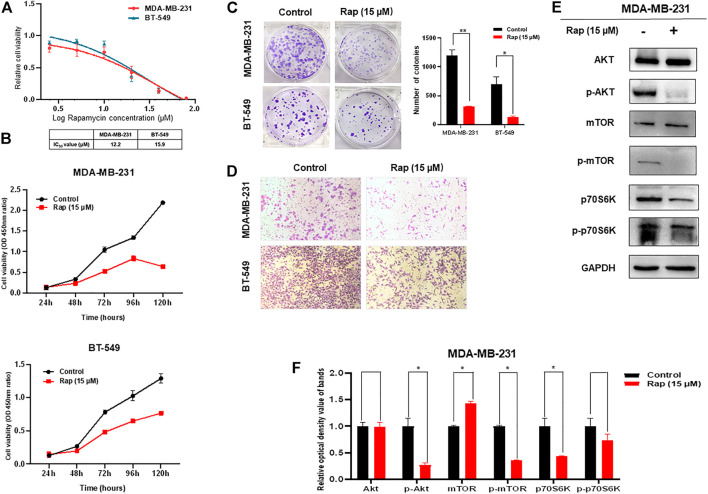
Effect of rapamycin on TNBC cells. **(A)** Viability of TNBC cells treated with varying concentrations of rapamycin **(B)** Proliferation of TNBC cells was inhibited by rapamycin in time-dependent manner **(C)** Colony formation of both MDA-MB-231 and BT-549 cells were suppressed by rapamycin treatment, analyzing in histogram **(D)** Motility of MDA-MB-231 and BT-549 cells was dramatically inhibited by rapamycin treatment **(E,F)** Rapamycin treatment decreased the activity of the AKT/mTOR signaling pathway. The quantified results from Western blot **(E)** were analyzed with statistical significance in histogram (F). *p < 0.05, **p < 0.01, ***p < 0.001.

The ability of rapamycin to inhibit growth of TNBC cells was also determined by CCK-8 assay, which showed IC50s of 12.2 μM for MDA-MB-231 and 15.9 μM for BT-549 cells (Figure 2A). Not surprisingly, the proliferation and colony formation of both MDA-MB-231 and BT-549 cells were suppressed by rapamycin treatment at the IC50 concentration (Figures 2B,C). The number of migratory cells in the transwell assay was lower in the rapamycin-treated group than that in the control (Figure 2D). As an mTOR inhibitor, rapamycin treatment of TNBC cells resulted in low activity of the AKT/mTOR signaling pathway, as demonstrated by decreased expression of p-AKT, p-mTOR, and p-p70S6K (Figures 2E,F).

### Combined Treatment of Itraconazole and Rapamycin Suppresses Triple-Negative Breast Cancer Cells Synergistically

**FIGURE 3:**
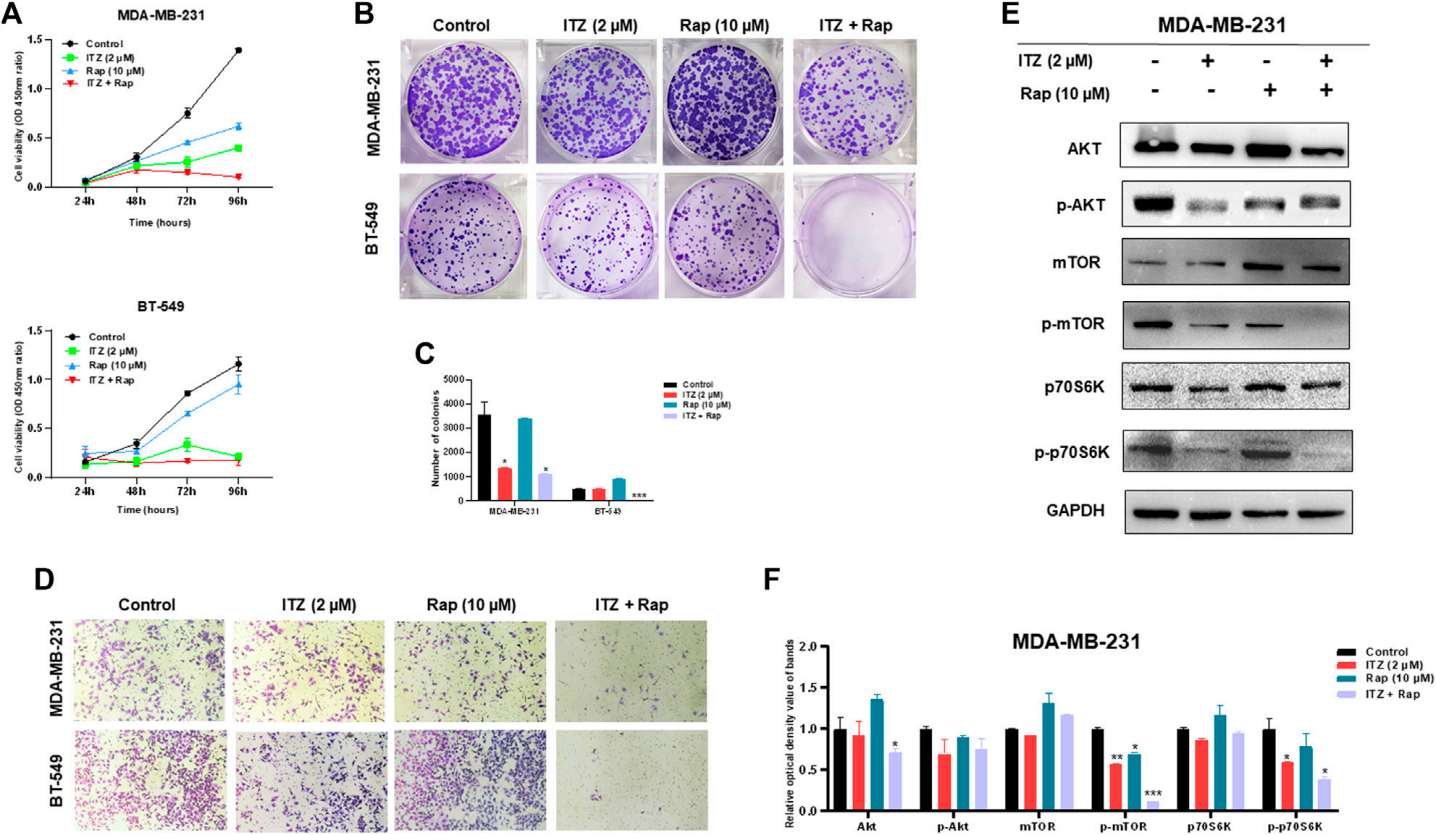
Combined effect of ITZ and rapamycin in TNBC cells. **(A,B)** Proliferation (A) and colony formation (B) of both MDA-MB-231 and BT-549 cells were suppressed by treatment with ITZ, rapamycin and ITZ + rapamycin **(C)** The quantified results of colony formation in TNBC cell treated with control, monotherapy and combined therapy **(D)** Motility of MDA-MB-231 and BT-549 cells was dramatically inhibited by treatment with ITZ, rapamycin and ITZ + rapamycin **(E)** AKT/mTOR signaling pathway activity was decreased by treatment with ITZ and rapamycin alone and combined **(F)** The quantified results from Western blot were analyzed with statistical significance in histogram. *p < 0.05, **p < 0.01, ***p < 0.001.

To investigate the potential function of combined ITZ with rapamycin in TNBC cells, CCK-8, colony formation and transwell assays were used to evaluate the malignant behavior of TNBC cells. In Figure 3A, the proliferation of both MDA-MB-231 and BT-549 cells was suppressed in both the ITZ- and rapamycin-treated groups, compared with the control groups, while the combined treatment inhibited the proliferation of TNBC cells dramatically compared to treatment with either drug alone. Consistently, colony formation was decreased by ITZ or rapamycin treatment alone, but the decline was even greater in the combined treatment groups than that in the groups treated with either drug alone (Figures 3B,C). Regarding the migratory ability of TNBC cells, transwell assays showed that the inhibition of TNBC cell motility was the most obvious in the combined treatment of ITZ and rapamycin rather than treatment with ITZ or rapamycin alone (Figure 3D).

The activity of the AKT/mTOR signaling pathway was also evaluated by the expression of biomarkers. In Figures 3E,F, although the expression level of AKT, mTOR, and p70S6K was not decreased following treatment with ITZ and rapamycin alone or in combination, their activated forms, i.e. p-mTOR, and p-p70S6K, were decreased in the single drug-treated groups, and were even lower in the combined treatment group.

### Itraconazole and Rapamycin Induce Triple-Negative Breast Cancer Cell Apoptosis Seperately

**FIGURE 4:**
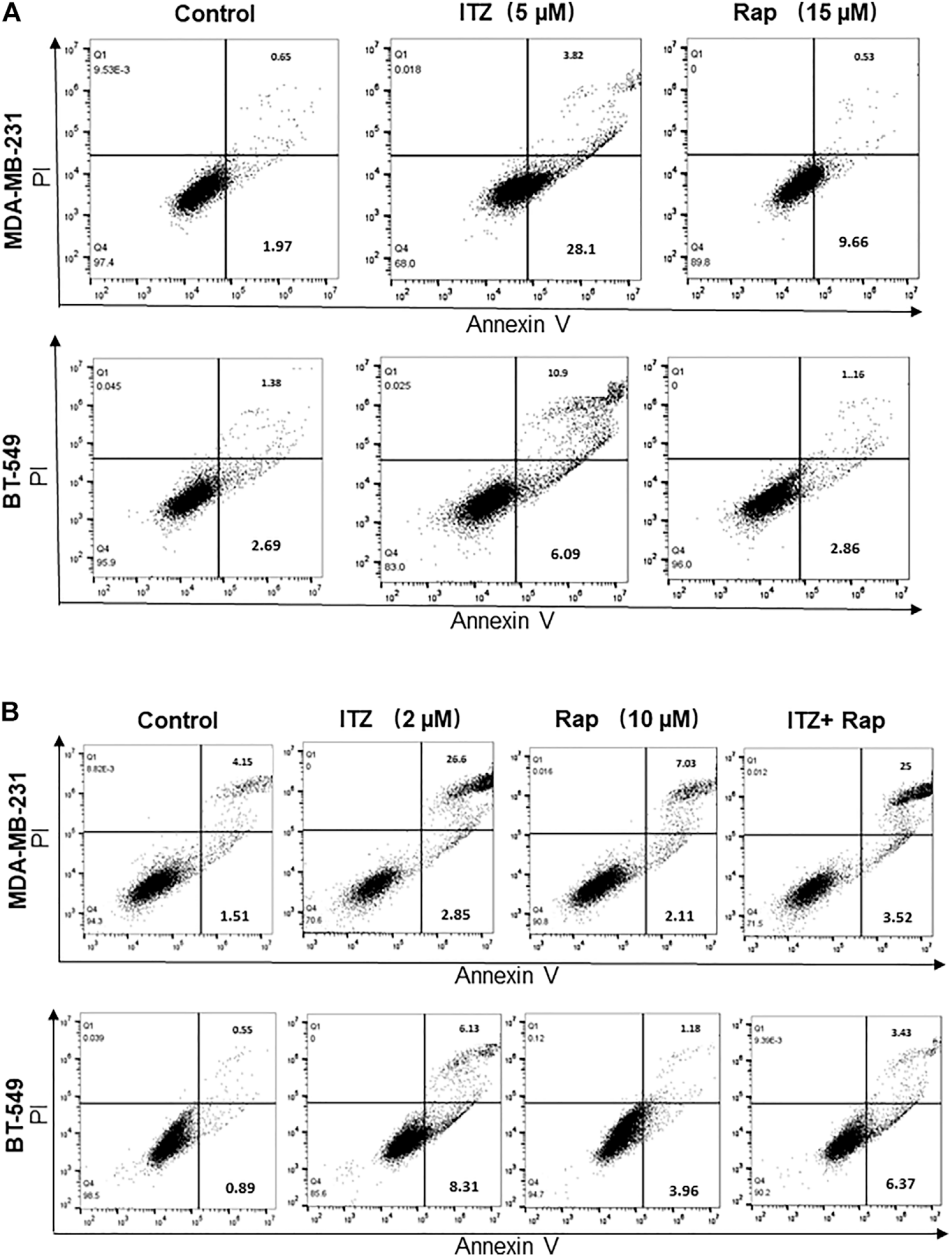
Apoptosis induced by ITZ and/or rapamycin in TNBC cells. **(A)** The percentage of apoptotic cells with or without ITZ or rapamycin treatment **(B)** The combined treatment of TNBC cells induced apoptosis without a synergistic effect.

In order to investigate the combined effect of ITZ and rapamycin in TNBC, the percentage of apoptotic cells was evaluated by flow cytometry after ITZ and rapamycin treatment. Apoptosis was induced by ITZ treatment and rapamycin treatment alone in MDA-MB-231 cells (Figure 4A), whereas for BT-549 cells, ITZ but not rapamycin induced apoptosis (Figure 4A).

For the combined treatment of ITZ and rapamycin, the cells were collected after a 48 h treatment and analyzed by flow cytometry. Although in the ITZ-treated group, apoptosis was induced accordingly, the percentage of apoptotic cells in the combined treatment group was not increased compared with the single drug groups (Figure 4B), indicating that the synergistic inhibition of ITZ and rapamycin on TNBC cells was not through inducing apoptosis.

### Synergistic Inhibition of Itraconazole in Combination With Rapamycin Is due to Cell Cycle Arrest

**FIGURE 5:**
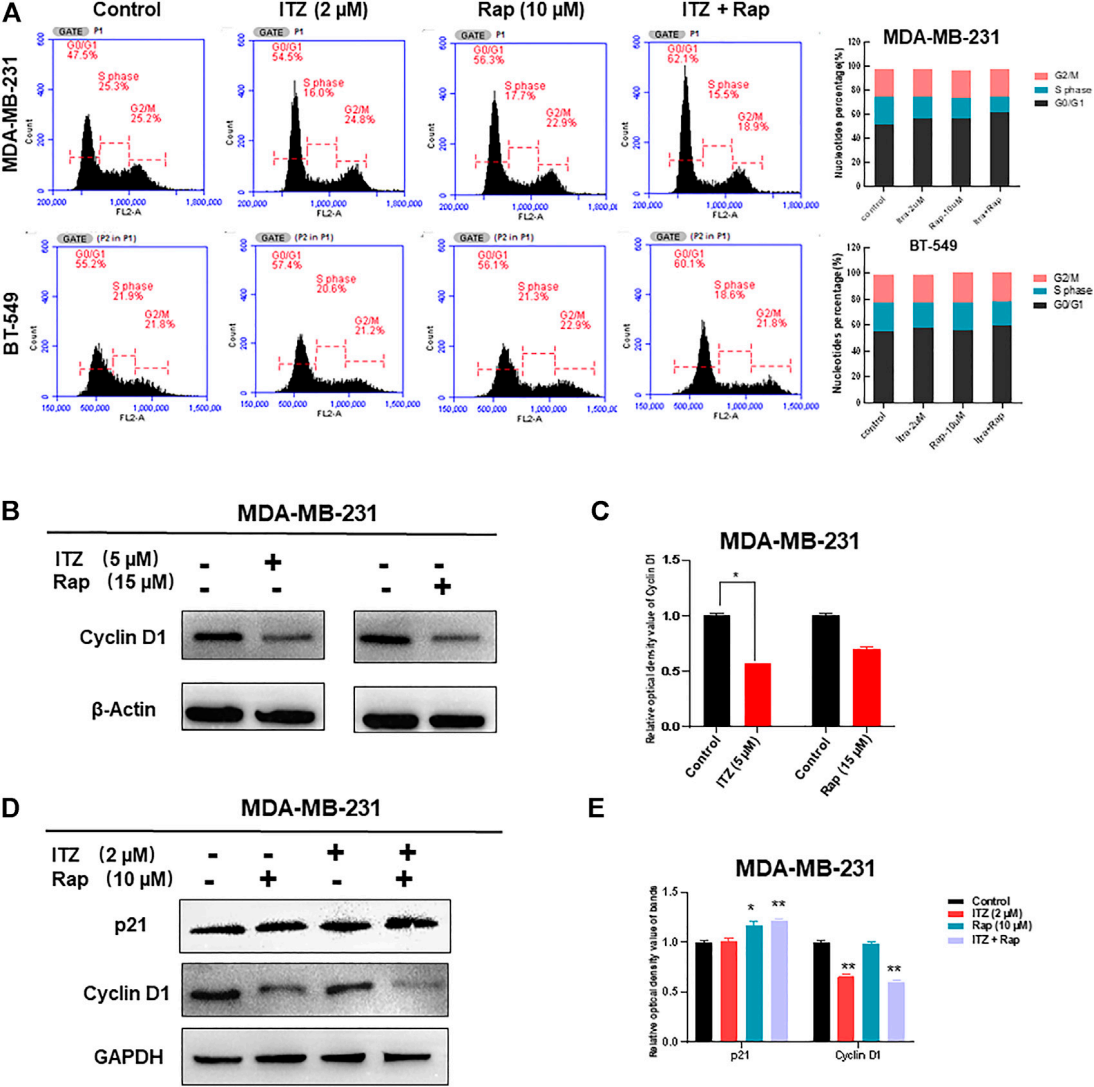
Cell cycle arrest caused by synergism between ITZ and rapamycin in TNBC cells. **(A)** ITZ and rapamycin caused cell cycle arrest in G0/G1 phase to inhibit the malignant behavior of TNBC cells **(B,C)** Expression of cyclin D1 was suppressed by ITZ (5 μM) or rapamycin (15 μM) treatment, analyzed in histogram (C) **(D,E)** Expression of cyclin D1 was decreased by the synergistic effect of ITZ (2 μM) combined with rapamycin (10 μM) in TNBC cells, whereas p21 accumulated in the combined group, analyzed in histogram (E). *p < 0.05, **p < 0.01, ***p < 0.001.

As apoptosis was not synergistically induced by ITZ and rapamycin in combination, the question arose as to how the proliferation and motility of TNBC cells was inhibited. Cell cycle analysis of TNBC cells was conducted to investigate the underlying mechanism. Interestingly, the proportion of TNBC cells in G0/G1 phase increased with ITZ and rapamycin treatment alone, but was especially high in the combined treatment group (Figure 5A).

Concomitant with cell cycle arrest in G0/G1 phase, the number of TNBC cells entering S and G2 phases was decreased accordingly. To confirm the synergistic effect of ITZ and rapamycin on blocking cell cycling, cell cycle-related proteins were detected by western blotting, which showed that treatment with ITZ (5 μM) or rapamycin (15 μM) alone suppressed the expression of cyclin D1, an indicator of G1/S phase transition (Figures 5B,C). Importantly, upon combined treatment with ITZ (2 μM) and rapamycin (10 μM), the expression of cyclin D1 was the lowest compared with control and single drug-treated groups (Figures 5D,E).

## Discussion

For patients with TNBC, chemotherapy has been the main therapeutic strategy due to the lack of targeted therapy. However, chemo-resistance, distant metastasis and relapse seriously affect the quality of life and survival of patients with TNBC (Shi et al., 2018). Drug combinations or drug repurposing have the potential to treat TNBC and/or reduce the amount of drug and related toxicity. Important signaling pathways, such as NF-κB, PI3K/Akt/mTOR, Notch 1, Wnt/β-catenin, and YAP, have been considered as therapeutic targets for patients with cancer (Lee et al., 2020). The current study proposes a novel combination of ITZ and rapamycin, both of which target the mTOR signaling pathway.

First, the optimal concentration used for TNBC cells was evaluated by CCK-8 assay. Interestingly, although both MDA-MB-231 and BT-549 cells are TNBC cell lines, the tolerance to ITZ and rapamycin are quite different with different IC50 values. For other types of breast cancer cell lines, the sensitivity to ITZ and rapamycin can be different based on the exposure time (Tengku Din et al., 2014; Ozates et al., 2021). Our results show that at the IC50 concentrations for ITZ and rapamycin, the proliferation and motility of both MDA-MB-231 and BT-549 cells are inhibited.

Cai *et al.* investigated the influence of different triazole antifungal drugs on the pharmacokinetics of cyclophosphamide, and all tested triazoles, including ITZ, increased the pharmacokinetics of cyclophosphamide in cancer patients, as well as the area under the plasma concentration-time curve of cyclophosphamide (Cai et al., 2020). Importantly, it has been reported that combination with ITZ treatment enhanced the toxicity due to chemotherapy in patients with Hodgkin’s lymphoma, causing severe myelosuppression and neurotoxicity after concurrent administration (Bashir et al., 2006). However, our combined treatment used lower concentrations of ITZ and rapamycin than the IC50, with the hypothesis that ITZ would enhance the cytotoxicity of rapamycin in TNBC cells. As expected, synergistic inhibition was observed to occur through suppressing the AKT/mTOR signaling pathway.

Further investigation focused on the mechanism of synergism in TNBC cells. Both apoptosis and cell cycle arrest are involved in this process. Flow cytometric analyses were conducted in this study. Interestingly, no enhanced induction of apoptosis was found upon combined treatment, indicating other mechanisms are involved in the inhibition of malignant behavior. Buczacki *et al.* demonstrated that ITZ could cause cycling and dormant cells to switch to global senescence in CRC models (Buczacki et al., 2018). Consistent with this, either ITZ or rapamycin treatment induced cell cycle arrest. Moreover, combined treatment with ITZ and rapamycin enhanced cell cycle arrest in TNBC cells through or partially p21 protein and decreasing cyclin D1 levels.

Nevertheless, attention also should be paid to side effects due to ITZ-enhanced toxicity of other compounds. In adult acute lymphoblastic leukemia, ITZ was found to enhance vindesine neurotoxicity (Chen et al., 2007). Recently, Foroughinia *et al.* reported vincristine-induced seizures potentiated by ITZ following chemotherapy of diffuse large B-cell lymphoma (Foroughinia et al., 2012), calling for attention to the need to evaluate the benefits and risks of patients before clinical use.

## Conclusion

Combination of ITZ with rapamycin exerts an antitumor effect on TNBC cells through arresting cell cycling in G0/G1 phase, rather than synergistically inducing apoptosis.

## Data Availability

The raw data supporting the conclusion of this article will be made available by the authors, without undue reservation.
